# Hyalinizing clear cell carcinoma of the sublingual gland: A case report and literature review

**DOI:** 10.1097/MD.0000000000039150

**Published:** 2024-07-26

**Authors:** Li Guan, Yuyang Tang, Zhenglin Yang, Lijuan Guo, Sen Yang

**Affiliations:** a School of Stomatology, Zunyi Medical University, Zunyi, China; b Department of Oral and Maxillofacial Surgery, Suining Central Hospital, Suining, China; c Department of Medical Cosmetology, Suining Central Hospital, Suining, China.

**Keywords:** case report, hyalinizing clear cell carcinoma, immunohistochemistry, salivary gland tumor, sublingual gland

## Abstract

**Rationale:**

Hyalinizing clear cell carcinoma (HCCC) of the salivary glands is a rare low-grade malignant tumor. This type of tumor is particularly uncommon in the sublingual glands.

**Patient concerns:**

A 57-year-old female with a mass on the left side of the floor of the mouth that had been present for 2 months. The computed tomography scan of the neck revealed a nodular abnormal density shadow in the left sublingual area, measuring approximately 2.6 cm × 1.9 cm.

**Diagnoses:**

Primary HCCC of the sublingual gland.

**Interventions:**

The patient underwent surgical treatment and reconstruction using a left anterolateral femoral free flap, which showed immunohistochemical positivity for CK 5/6, CK 7, CK (AE1/AE3), and Ki-67 (<5%), but negative for SMA and S-100.

**Outcomes:**

No recurrence was observed during the 12-month postoperative follow-up period.

**Lessons:**

The absence of characteristic clinical manifestations makes HCCC highly susceptible to misdiagnoses. This case presents a rare instance of HCCC in the sublingual gland, providing a reference for the clinical diagnosis and treatment of the disease.

## 1. Introduction

Hyalinizing clear cell carcinoma (HCCC) is a rare, low-grade malignant tumor that accounts for <1% of all salivary gland tumors.^[1]^ Diagnosis is challenging because of the similarity in the clinical manifestations and pathological features of highly malignant salivary gland tumors. Currently, diagnosis is mostly based on a combination of exclusion criteria. This paper reports the case of a patient admitted to our hospital with a primary HCCC of the sublingual gland. Relevant literature is discussed to provide a reference for the clinical diagnosis and treatment of this disease.

## 2. Case report

A 57-year-old female presented with a painless mass on the left floor of the mouth for 2 months duration. She had no history of smoking or alcohol consumption, tumors elsewhere in the body, or abnormalities on general examination. On specialist examination, an ovoid mass measuring 3.0 cm × 1.5 cm was palpated in the area of the left sublingual gland, which was hard, well-demarcated, poorly mobile, and caused no discomfort on palpation. A lymph node measuring approximately 1.0 cm × 0.8 cm was also palpable in the left submandibular region.

**Figure 1.**
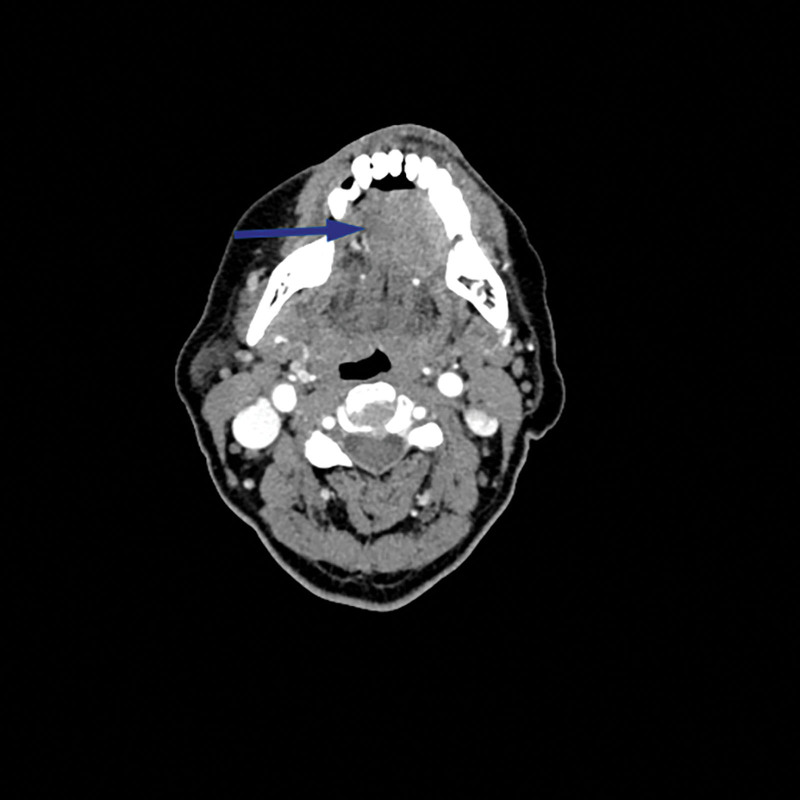
CT enhancement of the neck showing a left sublingual mass (blue arrow).

The computed tomography scan of the neck revealed a nodular abnormal density shadow in the left sublingual area, measuring approximately 2.6 cm × 1.9 cm, with indistinct borders. Enhanced computed tomography (Fig. 1) showed a more pronounced enhancement of the lesion, with no local central enhancement. Multiple small lymph nodes were observed in the bilateral neck interspaces, with the largest node located in the left submandibular region, measuring about 0.6 cm × 0.8 cm.

**Figure 2.**
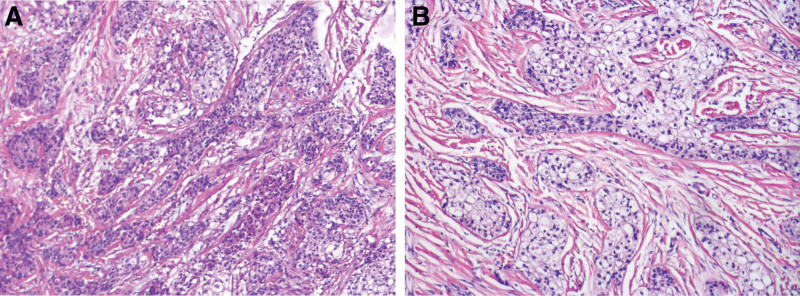
Histopathological manifestations (HE) (×100). (A) Preoperative biopsy; (B) postoperative pathology.

**Figure 3.**
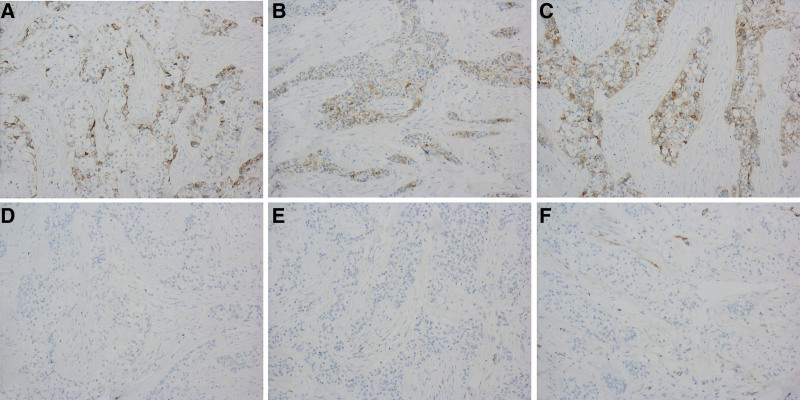
Immunohistochemical staining (×100). (A) CK 5/6 (+); (B) CK 7 (+); (C) CK (AE1/AE3) (+); (D) Ki-67 (+, <5%); (E) SMA (-); (F) S-100 (-).

Preoperative biopsy of the mass revealed a low-grade malignant tumor (Fig. 2A). Chest radiography and abdominal ultrasonography were performed to rule out metastasis, and the primary tumor was identified. Under general anesthesia, the patient underwent left supraclavicular lymph node dissection, left submandibular gland tumor resection, and left anterolateral femoral flap-free grafting. The surgical procedure was successful, and the patient recovered well postoperatively. Postoperative pathology revealed the presence of clear tumor cells and mesenchyme that were visible under a microscope (Fig. 2B). The tumor showed visible nerve invasion, but no invasion of the submitted jawbone or definite choroidal invasion. Four lymph nodes were not visible. Immunohistochemistry results were positive for cytokeratin (CK) 5/6, CK 7, CK (AE1/AE3), and Ki-67 (<5%), while SMA and S-100 were negative (Fig. 3). In conclusion, this patient was diagnosed with primary HCCC. No recurrence or distant metastasis was observed during the 12-month postoperative follow-up period.

## 3. Discussion

HCCC is a rare tumor that originates from the salivary glands. Clinical symptoms usually present as painless swelling and are more common in females than males, typically occurring between the ages of 50 and 80.^[2]^ Two-thirds of cases occur in the small salivary glands, mainly in the palate and tongue base.^[3]^ This tumor type can also occur in unusual locations, as reported in this case, the sublingual gland.

The tumor cells had a clear morphology and were surrounded by a transparent connective tissue matrix.^[4]^ Immunohistochemical staining was positive for cytokeratin (such as CK 5, CK 7, CK 14, and CK 19), while myoepithelial markers were negative (SMA, S-100, and MSA). Tumor cells are rich in glycogen and positive for PAS. The Ki-67 proliferation index is low.^[5]^ In some cases, S-100 expression may be positive, whereas in the same case, the above-mentioned immunohistochemistry may not be expressed simultaneously. This can lead to an overlap with other salivary gland tumors. The features that aid in the diagnosis of HCCC are as follows: (1) HCCC is more prevalent in the salivary glands of middle-aged women; (2) tumor cells exhibit small atypia, transparent cytoplasm, uniform and small nuclei, round or oval, and rarely appear to divide; (3) infiltrative changes are often observed at the edge of the tumor, with visible nerve invasion, but no vascular invasion^[6]^; and (4) the fusion of the EWSR1-ATF1 gene is considered specific to this tumor.^[7]^ After reviewing this case and combining the results of the 2 pathological sections, it was found that the patient was a middle-aged woman who sought a diagnosis of a painless mass at the bottom of her mouth. During surgery, neurological invasion was observed but vascular invasion was not detected. Furthermore, immunohistochemistry revealed positive expression of CK and PAS, negative expression of the muscle epithelial marker SMA, and low expression of Ki-67, which confirmed the diagnosis of clear cell carcinoma.

HCCC is generally considered to be a low-grade malignancy with a good prognosis. Misdiagnosis of highly malignant tumors of the head and neck (such as mucoepidermoid carcinoma, squamous cell carcinoma, and epithelial myoepithelial carcinoma) may lead to excessive and unnecessary treatment.^[8]^ In this case, HCCC of the sublingual gland was easily diagnosed as myoepithelial carcinoma because both HCCC and myoepithelial carcinoma are rare and can be composed of clear cells, which may overlap to some extent on histopathology. Therefore, we focused on distinguishing between these 2. (1) Morphologically, myoepithelial carcinoma has a typical ductal structure covered by a double-layered epithelium, with myoepithelial cells surrounding tubular eosinophils; HCCC tumor cells have a simple morphology and a large amount of transparent substance in the stroma. (2) Immunohistochemically, the expression of S-100 protein and myoepithelial markers was significantly positive in myoepithelial carcinoma; HCCC tumor cells were CK-positive, PAS-positive, and myoepithelial marker negative. In addition, the EWSR1 rearrangement has specific specificity for clear cell salivary gland carcinoma. Therefore, a combination of thorough clinical evaluation, histology, immunohistochemical staining, and molecular genetic features can to some extent avoid misdiagnosis of HCCC.^[9]^

Currently, no evidence-based treatment options are available for HCCC. The most effective treatment involves a combination of wide local excision and regional lymph node dissection. Although clear cell carcinoma of the salivary glands is considered a low-grade malignancy, a recent report by Desai et al showed that of the 202 cases published between 1983 and 2020, 13.9% had local and distant metastases and 18.8% had local tumor recurrence. This suggests that the malignancy is not as inert as previously thought but still has a good overall prognosis.^[1]^ Postoperative radiotherapy is recommended for patients with locoregional metastasis. Recurrence occurs after treatment, and risk factors for recurrence may include the presence or absence of focal necrosis at the time of diagnosis and the presence or absence of local metastases, and long-term postoperative clinical and radiological follow-up is recommended.^[10]^

In conclusion, HCCC is a rare disease that has not been widely studied and it is challenging for clinicians to diagnose rare diseases that occur in unusual locations. Although immunohistochemistry for HCCC is nonspecific and molecular genetics are not unique, combining thorough clinical history, immunohistochemistry, and molecular genetic features can help clinicians make an accurate diagnosis. There are no specific treatment guidelines for HCCC, and treatment is preferred over wide local excision combined with regional lymph node dissection, with postoperative radiotherapy targeted at patients with locoregional metastases who have better prognosis. Long-term follow-up of this patient may help us to further understand the prognostic factors and treatment planning and better to understand this rare malignancy of the sublingual gland.

## Author contributions

**Data curation:** Yuyang Tang.

**Resources:** Zhenglin Yang.

**Supervision:** Lijuan Guo.

**Visualization:** Li Guan.

**Writing – original draft:** Li Guan, Yuyang Tang.

**Writing – review & editing:** Sen Yang.
